# Novel pathogenic mutations in *C1QTNF5* support a dominant negative disease mechanism in late-onset retinal degeneration

**DOI:** 10.1038/s41598-017-11898-3

**Published:** 2017-09-22

**Authors:** Chloe M. Stanton, Shyamanga Borooah, Camilla Drake, Joseph A. Marsh, Susan Campbell, Alan Lennon, Dinesh C. Soares, Neeru A. Vallabh, Jayashree Sahni, Artur V. Cideciyan, Baljean Dhillon, Veronique Vitart, Samuel G. Jacobson, Alan F. Wright, Caroline Hayward

**Affiliations:** 1 Medical Research Council Human Genetics Unit, Medical Research Council Institute of Genetics and Molecular Medicine, University of Edinburgh, Edinburgh, United Kingdom; 2 Medical Research Council Centre for Regenerative Medicine, University of Edinburgh, Edinburgh, United Kingdom; 3 Princess Alexandra Eye Pavilion, Edinburgh, United Kingdom; 4 St. Paul’s Eye Unit, Royal Liverpool Hospital, Liverpool, United Kingdom; 5 Department of Eye and Vision Sciences, University of Liverpool, Liverpool, United Kingdom; 6 Roche Pharma Research and Early Development, Roche Innovation Center Basel, F. Hoffmann-La Roche Ltd, Basel, Switzerland; 7 Scheie Eye Institute, University of Pennsylvania, Philadelphia, Pennsylvania USA; 8 Centre for Clinical Brain Sciences, School of Clinical Sciences, University of Edinburgh, Edinburgh, United Kingdom

## Abstract

Late-onset retinal degeneration (L-ORD) is a rare autosomal dominant retinal dystrophy, characterised by extensive sub-retinal pigment epithelium (RPE) deposits, RPE atrophy, choroidal neovascularisation and photoreceptor cell death associated with severe visual loss. L-ORD shows striking phenotypic similarities to age-related macular degeneration (AMD), a common and genetically complex disorder, which can lead to misdiagnosis in the early stages. To date, a single missense mutation (S163R) in the *C1QTNF5* gene, encoding C1q And Tumor Necrosis Factor Related Protein 5 (C1QTNF5) has been shown to cause L-ORD in a subset of affected families. Here, we describe the identification and characterisation of three novel pathogenic mutations in *C1QTNF5* in order to elucidate disease mechanisms*. In silico* and *in vitro* characterisation show that these mutations perturb protein folding, assembly or polarity of secretion of C1QTNF5 and, importantly, all appear to destabilise the wildtype protein in co-transfection experiments in a human RPE cell line. This suggests that the heterozygous mutations in L-ORD show a dominant negative, rather than a haploinsufficient, disease mechanism. The function of C1QTNF5 remains unclear but this new insight into the pathogenetic basis of L-ORD has implications for future therapeutic strategies such as gene augmentation therapy.

## Introduction

Recent advances in retinal gene therapy emphasise the importance of understanding pathogenetic mechanisms in inherited retinal dystrophies^1^. One such disorder is late-onset retinal degeneration (L-ORD), a rare autosomal dominant disorder with onset in the fifth or sixth decade, associated with night blindness, progressive central and peripheral visual field loss and choroidal neovascularization (CNV)^2–4^. The clinical and pathological resemblance to age-related macular degeneration (AMD) has been emphasised since both disorders show early dark adaptation delay, focal and diffuse sub-retinal pigment epithelial (RPE) deposits resembling basal laminar drusen, sharply demarcated areas of RPE and later choroidal atrophy and CNV^5^.

All previously reported cases of L-ORD result from a single S163R founder mutation in the *C1QTNF5* gene encoding C1QTNF5, a 24 kDa secreted and membrane-associated protein that is strongly expressed in RPE cells^5^. The function of C1QTNF5 is unknown but it contains a signal peptide, a short collagen domain with 23 Gly-X-Y repeats and a globular complement 1q/tumor necrosis factor-like (gC1q) domain^5,6^. The *C1QTNF5* gene is contained within the 3′ untranslated region of another gene, *MFRP* (Membrane Type Frizzled Related Protein)^5^, which is also highly expressed in RPE and associated with causal mutations in nanophthalmos^7^, retinitis pigmentosa, foveoschisis and optic disc drusen^8–10^.

C1QTNF5 is thought to be a multimeric protein with native trimeric and high molecular weight forms^6^. A crystal structure of the trimeric gC1q domain^11,12^, suggested that the high molecular weight form is an octadecamer, consisting of a bouquet-like arrangement of six gC1q trimers tethered by their collagenous stalks, resembling other C1q/TNF family members such as C1q and adiponectin. These studies further suggested that the S163 residue forms an intermolecular hydrogen bond network involved in tethering the globular gC1q heads together into a stable trimer, which is weakened by the R163 founder mutation^11,12^.

We now report the identification and characterisation of three novel C1QTNF5 mutations associated with L-ORD, each of which supports a dominant negative disease mechanism in L-ORD, which has important implications for future therapies.

## Results

### Clinical evaluation of L-ORD family members

**Figure 1:**
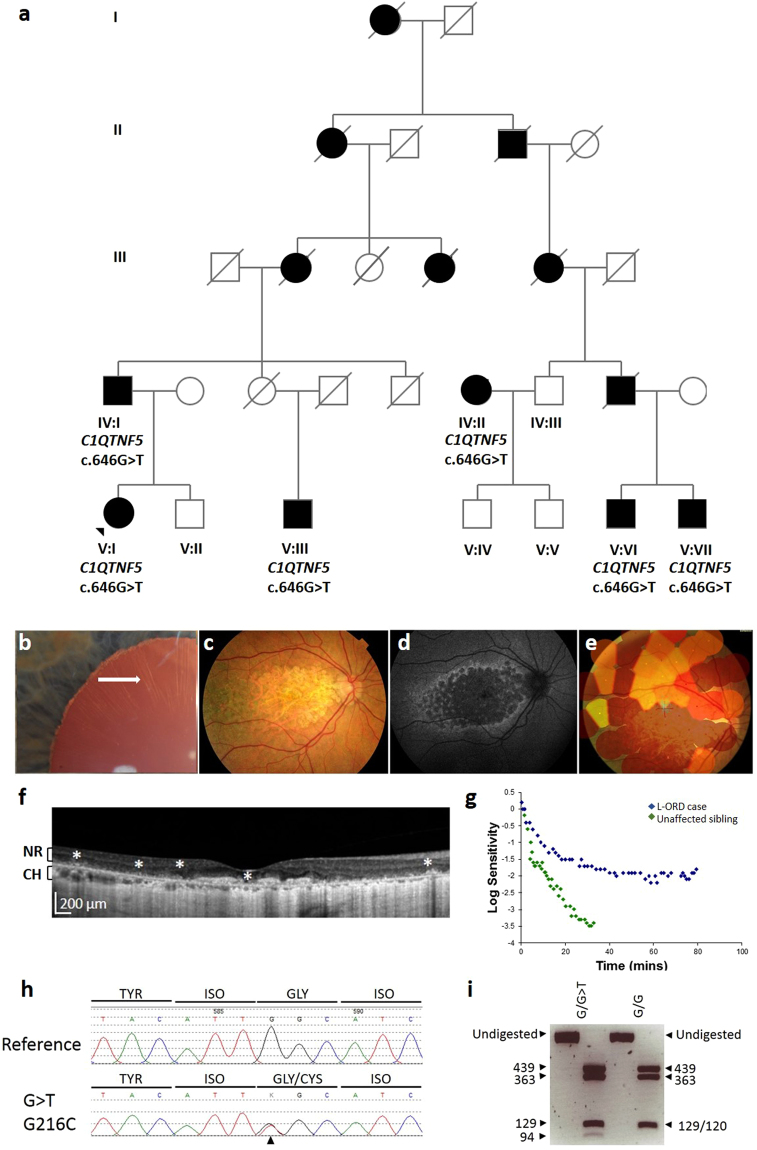
Pedigree, genotype and phenotype of family 1. (**a**) L-ORD family 1 is a multi-generation pedigree showing autosomal dominant inheritance. Squares represent males, circles represent females. Shaded shapes indicate an affected individual. Clinical phenotyping of the proband V:I. (**b**) Anterior segment slit lamp photograph shows long-anterior zonules (white arrow) encroaching beyond the pupil margin in a dilated pupil. (**c**) Colour fundus photography shows areas of central atrophy affecting the macula and (**d**) Fundus autofluorescence imaging shows areas of central scalloped hypoautofluorescence matching the areas of atrophy surrounded by a cuff of increased autofluorescence. (**e**) Microperimetry interpolated plot superimposes areas of retinal sensitivity on a colour fundus image. Areas of good sensitvity vary from green to yellow to red for poor sensitivity. Areas with no sensitivity have no polygons. The image shows non-detectable sensitivity at the macula for the patient. (**f**) Hyperreflective deposits deep to the neural retina, indicated by asterisks, are observed in the macula in an OCT scan of the proband. NR, neural retina; CH, choroid. (**g**) Dark adaptometry of the proband (L-ORD case) shows that recovery of sensitivity after light exposure is slow and final sensitivity is still reduced at 80 minutes, compared to the normal, rapid recovery of sensitivity in an unaffected sibling. (**h**) Sequence analysis of *C1QTNF5* exon 3 revealed a heterozygous c.646G > T transversion, resulting in a glycine to cysteine mutation at position 216 in the L-ORD case (lower panel) compared to an unaffected relative (upper panel). (**i**) HpyCh4V restriction digest confirms the c.646 G > T mutation by generating fragments of 439, 363, 129 and 120 bp in homozygous G (p.G216) samples, and 439, 363, 129, 94 and 26 bp for the mutant G > T (p.C216) allele.

The proband (V:1) in family 1 (Fig. 1a) was affected with L-ORD, with central visual loss in the sixth decade, and the characteristic clinical features on ophthalmic examination (Fig. 1). The features included long-anterior zonules (Fig. 1b), central atrophy affecting the macula (Fig. 1c–e), loss of the RPE in areas of atrophy, thickening of the sub-RPE layer and choroidal thinning (Fig. 1f), and abnormal dark adaptation, compared to an unaffected sibling V:II (Fig. 1g). Additional family members in generation IV and V of the pedigree described varied symptomatology (Fig. 1a, Table 1). Patient V:III had the most advanced disease, with early drusenoid changes in the peripheral macula followed by rapid progression of dark adaptation delay, central and then peripheral field loss. At age 72, he was already completely blind, and was noted to have an atrophic retina which clinically appeared like end-stage retinitis pigmentosa. By contrast, V:VI and V:VII showed a significant delay in dark adaptometry, an early phenotypic marker of L-ORD, but had minimal retinal changes, visual acuities of 6/6, and reported no loss of central vision in their sixth decade.

**Table 1:** Summary of Clinical and Genetic Features of Individuals with L-ORD.

Family	Sample ID	Genotype	Protein	Status	Age of onset	Dark adaptation delay	Fundus appearance		
							drusenoid deposits	RPE atrophy	macular degeneration
1	IV:I	Heterozygous c.646G>T	p.G216C	Affected	late 50s	yes	yes	yes	yes
	IV:II	Heterozygous c.646G>T	p.G216C	Affected	early 50s	yes	yes	yes	yes
	V:I	Heterozygous c.646G>T	p.G216C	Affected	mid-60s	yes	yes	yes	yes
	V:III	Heterozygous c.646G>T	p.G216C	Affected	late 50s	yes	yes	yes	yes
	V:VI	Heterozygous c.646G>T	p.G216C	Affected	mid-60s	yes	yes	no	no
	V:VII	Heterozygous c.646G>T	p.G216C	Affected	mid-60s	yes	yes	no	no
2	II:I	Heterozygous c.562C>A	p.P188T	Affected	mid-40s	yes	yes	yes	yes
	II:II	Heterozygous c.562C>A	p.P188T	Affected	mid-60s	yes	yes	yes	yes
	II:III	Heterozygous c.562C>A	p.P188T	Affected	late 50s	yes	yes	no	no
3	III:I	Heterozygous c.489C>A	p.S163R	Affected	mid-60s	yes	yes	yes	yes

**Figure 2:**
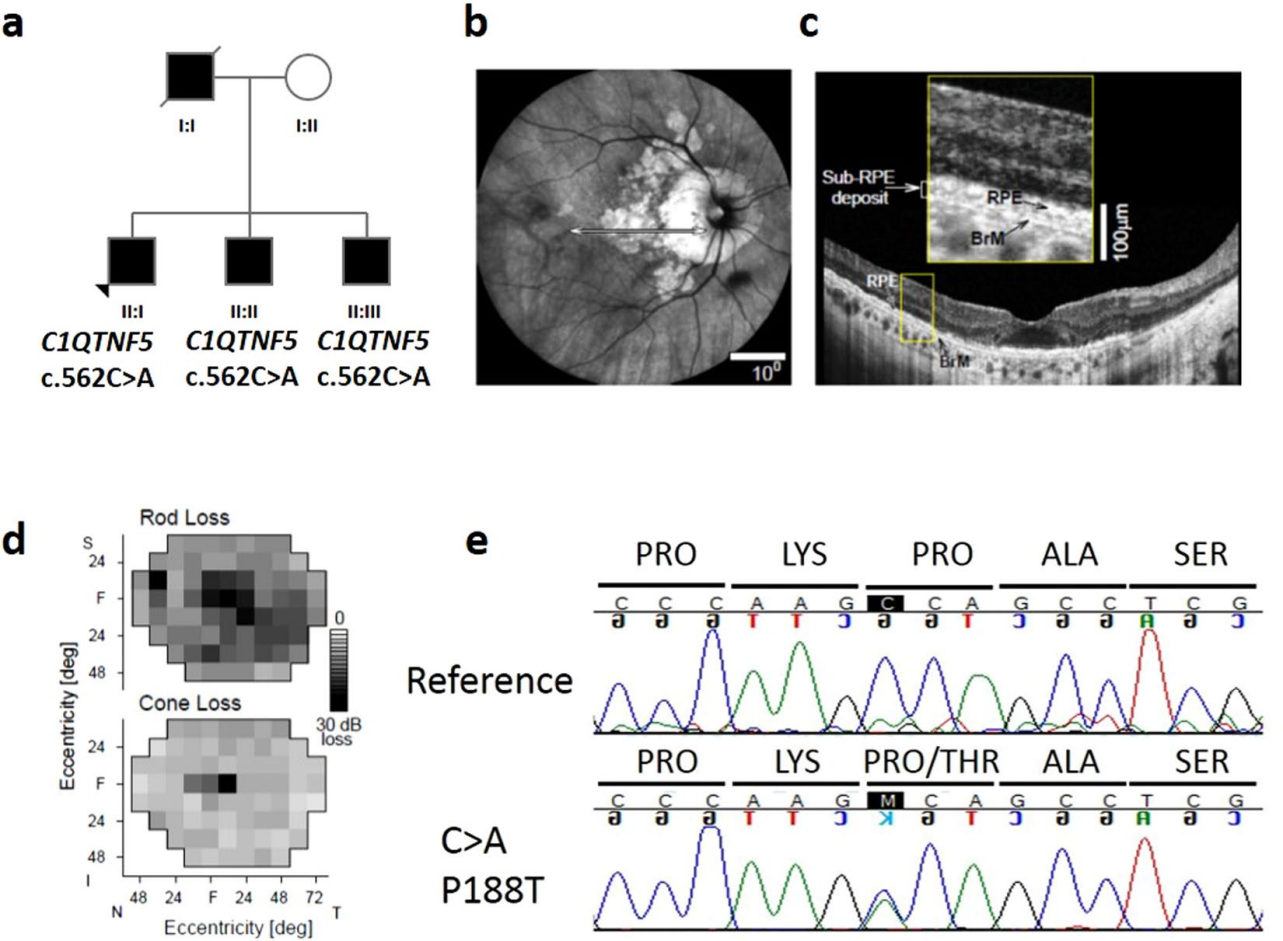
Pedigree, genotype and phenotype of family 2. (**a**) L-ORD family 2, in which affected family members are heterozygous for the normal and mutant p.P188T allele. Squares represent males, circles represent females. Shaded shapes indicate an affected individual. (**b**) *En face* near infrared reflectance image of right eye of proband showing circumpapillary and macular atrophic lesions. (**c**) OCT scan across the macula (defined in panel b by horizontal arrow) illustrating some preserved outer nuclear layer and sub-RPE deposits (inset, magnified part of scan labeling RPE and Bruch’s membrane, BrM). (**d)** Rod sensitivity loss (500 nm, dark-adapted) and cone sensitivity loss (600 nm, light adapted) visual field maps of the right eye of the proband. (**e**) Sequence analysis of *C1QTNF5* exon 3 revealed a c.562 C > A transversion, resulting in a proline to threonine mutation in the L-ORD case (lower panel) compared to the unaffected relative (upper panel).

The proband (II:I) in family 2 (Fig. 2a) denied any vision problems until his mid-40′s when he noticed difficulty adapting to darkness after a light exposure. Night vision became abnormal over the next decade until use of a flashlight was needed to walk in dimly lit surroundings. By age 60, there was decreased central vision and visual acuity, previously 6/6 in both eyes, became 6/12 (right eye) and 6/9 (left eye). Areas of chorioretinal atrophy around the optic nerve head extended to near the fovea (Fig. 2b). Optical coherence tomography showed preserved foveal outer nuclear layer (ONL) but decreasing ONL with increased eccentricity; there were sub-RPE deposits across the macula (Fig. 2c). Rod-mediated sensitivity across the visual field was reduced by an average of 16 dB; cone-mediated sensitivity loss was 9 dB (Fig. 2d). Among other affected family members was the deceased father (I:I) who lost central vision at age 60; vision deteriorated rapidly over the next decade and there was need for a cane and then guide dog. There were two affected brothers: a 64 year old (II:II) with visual acuities reduced to 6/60 in both eyes, peripheral visual field losses and circular areas of chorioretinal atrophy extending across the macula and pigmentary retinopathy peripherally. A younger brother (II:III), at age 57, had visual acuities of 6/6 in both eyes, complaints of night vision disturbances and drusenoid deposits across the macula. The family carried the diagnosis of retinitis pigmentosa but this was later changed to late-onset retinal degeneration.

**Figure 3:**
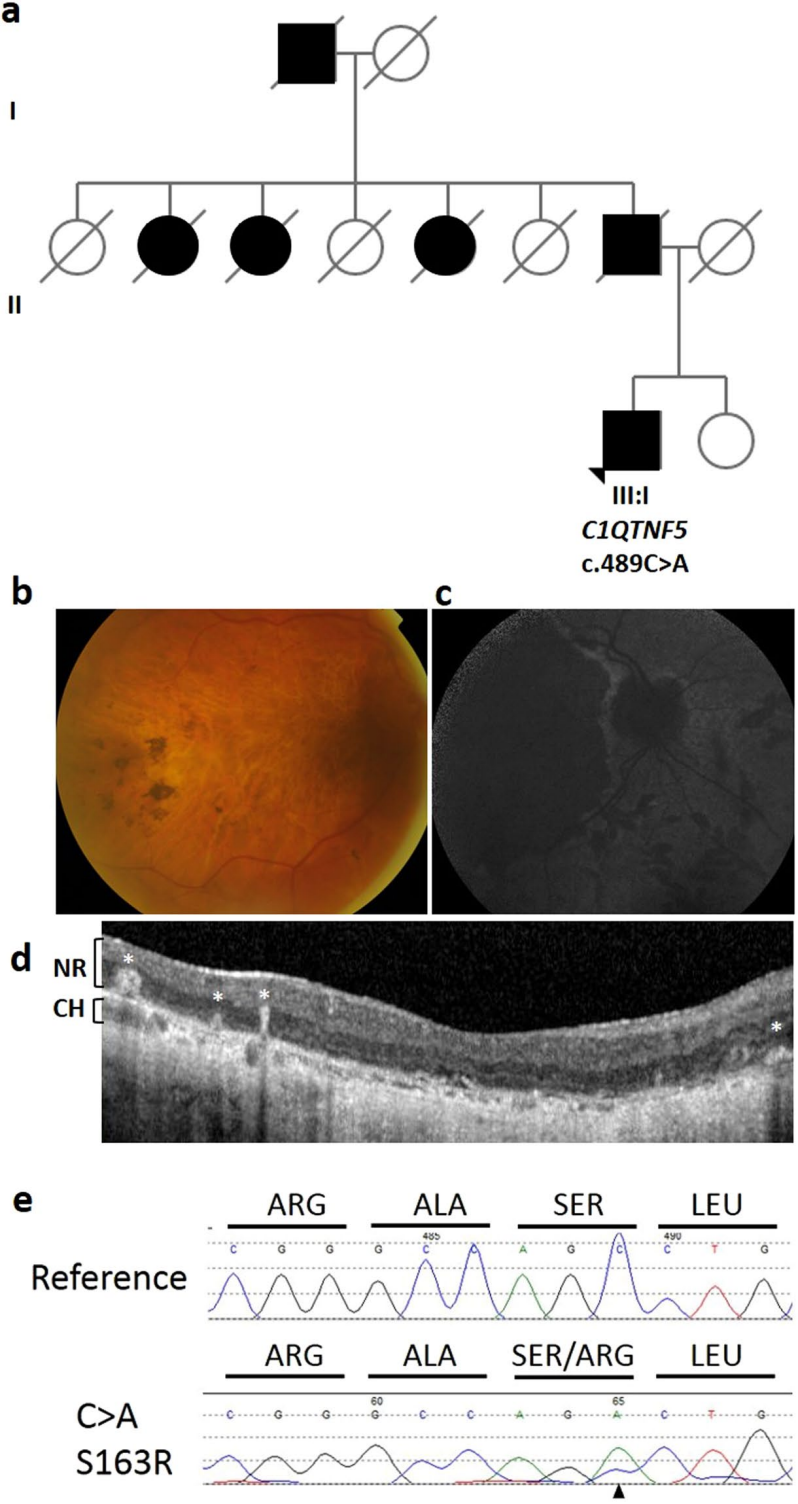
Pedigree, genotype and phenotype of family 3. (**a**) L-ORD family 3 is a multi-generation pedigree showing autosomal dominant inheritance. Squares represent males, circles represent females. Shaded shapes indicate an affected individual. Clinical phenotyping of the proband III:I (**b**) Colour fundus photography shows areas of central retinal atrophy. (**c**) Fundus autofluorescence imaging shows areas of hypoautofluorescence matching the areas of atrophy. (**d**) Hyperreflective deposits within and deep to the neural retina, indicated by white asterisks, are observed in the macula in an OCT scan of the proband. NR, neural retina; CH, choroid. (**e**) Sequence analysis of *C1QTNF5* exon 3 revealed a heterozygous c.489 C > A transversion, resulting in a serine to arginine mutation at position 163 in the L-ORD case (lower panel) compared to an unrelated, unaffected individual (upper panel).

Finally, we identified 23 unrelated suspected L-ORD cases presenting with characteristic features of the disease. One of these individuals was part of a multi-generational family affected by late-onset vision loss, inherited in an autosomal dominant manner. In this family (Fig. 3a), the affected paternal grandfather, father and aunts of the proband were deceased and so could not be examined or genotyped. The proband of family 3 (III:I) had a more than 15 year history of delayed dark adaptation, increasing nyctalopia and central and peripheral field loss. Visual acuity reduced to 1/60 in the right eye and hand movement sensitivity only in the left eye. Fundus imaging revealed the L-ORD phenotype, including long anterior zonules, atrophic macula, scalloped midpheripheral lesions with pigmentation, retinal vascular attenuation and OCT appearance showing loss of RPE and overlying outer retinal atrophy (Fig. 3b–d).

### Identification of novel pathogenic mutations in *C1QTNF5*

In two L-ORD families, the family history was consistent with an autosomal dominant pattern of inheritance (Figs 1–2). The probands were genotyped for the S163R mutation using *BstNI* restriction digestion, and were found to be homozygous for the wildtype, ancestral (S163) allele at this position, suggesting genetic heterogeneity in L-ORD. The entire coding region of *C1QTNF5*, located in chromosomal region 11q23, was then sequenced, and a novel heterozygous variant was identified in each family. In family 1, a c.646G > T transversion was identified in exon 3 of the proband and in 6 affected family members, but not in 4 unaffected family members (Fig. 1h). This variant results in a non-synonymous amino acid substitution p.G216C. The substitution creates a new *HpyCH4V* restriction site, which was used to confirm the genotypes obtained by direct sequencing in the L-ORD family (Fig. 1i). The c.646G > T p.G216C variant segregates with the disease in this family (Fig. 1a). In family 2 (Fig. 2a), a heterozygous c.562C > A transversion was identified in exon 3 of *C1QTNF5*, resulting in the missense substitution p.P188T (Fig. 2e). This variant segregated with disease in three affected siblings and was not observed in an unaffected parent. In light of the resultant allelic heterogeneity in L-ORD, we sequenced *C1QTNF5* in 23 unrelated suspected L-ORD cases that did not carry the S163R mutation, and identified a c.489C > A transversion in a single affected individual. This individual had a family history of L-ORD (Fig. 3a), but we were unable to obtain additional samples in order to verify the segregation of this variant with disease. Interestingly, the c.489C > A transversion (Fig. 3c) affects the same nucleotide as the L-ORD founder mutation (c.489C > G) described previously^5^ and results in the same missense amino acid substitution, p.S163R. Restriction enzymes were not available to verify the sequence changes for c.562C > A or c.489C > A. No additional disease-causing mutations were identified in *C1QTNF5*. None of the three variants identified in L-ORD cases were recorded in the ExAC database (http://exac.broadinstitute.org/), including rare variants identified by exome sequencing of 60,706 individuals^13^ (Supplementary Tables S1, S2).

### Comparative and structural studies of mutant C1QTNF5

**Figure 4:**
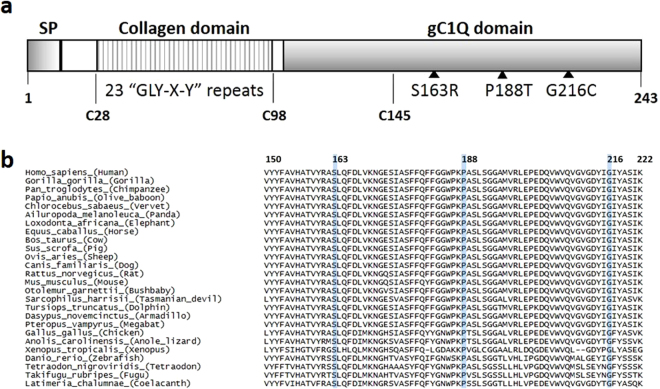
C1QTNF5 protein domains and orthologous sequence alignment. (**a**) Schematic structure of human C1QTNF5 protein. Signal peptide, SP (amino acids 1–15), collagen domain containing 23 Gly-X-Y repeats (amino acids 30–98), and the gC1q domain (amino acids 99–243), cysteine residues, and the positions of three novel pathogenic mutations are shown. (**b**) Sequence conservation of L-ORD mutations in orthologous C1QTNF5 protein sequences. Orthologous sequences were obtained from ensembl, and aligned using ClustalOmega. Amino acid numbering is relative to human C1QTNF5. S163, P188 and G216 are shown in blue, and are completely conserved throughout mammals, birds, fish and reptiles.

*C1QTNF5* encodes a member of the C1q/tumor necrosis factor superfamily, with characteristic collagen and gC1q domains (Fig. 4a). Sequence alignment of C1QTNF5 and orthologous genes in other species showed that S163, P188 and G216 are each fully conserved in mammals, birds, reptiles and fish (Fig. 4b). This is supported by positive PhyloP scores, and PhastCons scores of 1, indicating a high level of conservation at the individual base, amino acid and other conserved elements (Supplementary Table S1).

**Figure 5:**
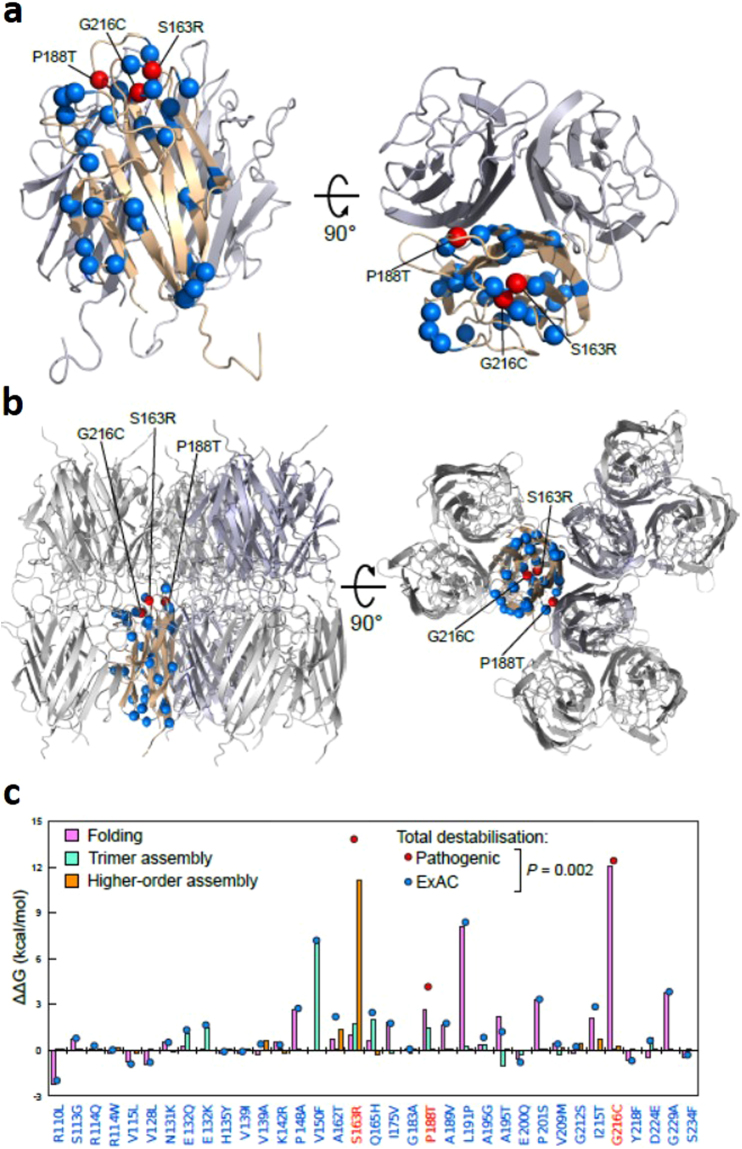
Structural modelling of the impact of variants in the gC1Q domain of C1QTNF5 upon folding and assembly of the monomer, trimer and octadecamer. (**a**) Structure of the C1QTNF5 gC1q domain homotrimer (PDB ID: 4F3J)^11^, with the locations of the pathogenic (red) and ExAC missense variants (blue) highlighted. (**b**) Structure of the C1QTNF5 gC1q domain 18-mer (PDB ID: 4NN0)^12^, with the locations of the pathogenic (red) and ExAC missense variants (blue) highlighted. (**c**) Comparison of the predicted effects on protein folding (pink) and assembly of trimers (green) and higher-order complexes (orange) for the pathogenic and ExAC amino acid substitutions. The total destabilisation, equal to the sum of the effect on folding and assembly, is shown as a red or blue dot for each substitution. The pathogenic substitutions are significantly more destabilizing than those in ExAC (*p* = 0.002, Wilcoxon rank-sum test).

The impact of missense amino acid changes identified in L-ORD families, and in the ExAC database, on the structure and function of C1QTNF5 was assessed using the online tools MutationTaster^14^, PROVEAN^15^, SIFT^16^ and PolyPhen2^17^ (Supplementary Table S1). Amino acid changes were classified as deleterious, damaging or disease-causing, depending on the probability score calculated by the specific algorithm based on sequence conservation, homology and structural information. As reported by others^18^, there were discrepancies between the predictions obtained across the different analysis tools – including S163R, a known pathogenic mutation that has severe effects on protein structure and function (Supplementary Table S1). To overcome these discrepancies, we therefore sought to more directly assess the effects of impacts of the new variants identified in L-ORD patients using an *in silico* mutagenesis approach. Using the crystal structure of the C1QTNF5 gC1q domain trimer (Fig. 5a), as well as the higher-order 18-mer that could modelled from contacts present in the crystal structure^12^ (Fig. 5b), we were able to computationally model the effects of each mutation on the overall protein stability. We could separate the effects of each mutation into the impact upon folding of the monomeric subunits, the assembly into trimers, and the assembly into 18-mers. The structure includes only amino acids 103–243^11,12^, and so does not include the collagen domain of full-length C1QTNF5 (amino acids 30–98, Fig. 4a).

Of the 35 amino acid substitutions, reported either in the ExAC database or in L-ORD families, that could be mapped onto the gC1q structure, the L-ORD mutations resulted in 3 of the 5 most energetically unfavourable alterations to protein structure and/or assembly, consistent with a pathogenic role in disease (Fig. 5, Supplementary Table S2). The G216C substitution was predicted to be the most deleterious using prediction programs PolyPhen2 (probably damaging, probability 1.0), PROVEAN (Deleterious, Score −8.62), SIFT (Damaging, score 0.0) and Mutation Taster (disease-causing, probability 0.999; Supplementary Table S1). Protein stability calculations predicted that this mutation is highly energetically unfavourable, resulting in a calculated total free energy difference (ΔΔG) of 12.4 kcal/mol, compared to the wild-type protein (Supplementary Table S2). This reflects a severely reduced stability of the protomer (ΔΔG 12.0 kcal/mol), resulting from steric clashes with the adjacent amino acids V159, A162, S163 and L164 within the monomer (Fig. 5a). By contrast, if the G216C mutant protein is stable enough to assemble into a trimer, the substitution is predicted to have essentially no impact on any intermolecular interactions in the trimer or 18-mer, since G216 does not form any interface contacts with other subunits (Fig. 5).

There was a discrepancy between mutation predictions using the various online tools for the p.P188T mutation identified in L-ORD family 2. This variant was predicted with high confidence to be probably damaging (PolyPhen2), disease-causing (Mutation Taster), neutral (PROVEAN) or tolerated (SIFT) (Supplementary Table S1). Our computational modelling of protein stability predicts that the substitution of proline to threonine at this position is energetically unfavourable (total ΔΔG 4.1 kcal/mol), with the strongest contribution to this due to the disruption of intramolecular interactions in the protomer (ΔΔG 3.6 kcal/mol), and assembly of the trimer affected to a lesser degree, despite the position of P188 at the interface between monomers in the complex (Fig. 5a–c).

The S163R missense mutation, previously described in several studies as being pathogenic in L-ORD, also gave rise to discrepant predictions regarding the consequences of the mutation using available online tools, being classified as neutral by PROVEAN, but damaging or disease-causing by SIFT, PolyPhen2 and Mutation Taster (Supplementary Table S1). *In silico* modelling predicted that S163R would alter protein stability. Interestingly, our *in silico* modelling predicts that this mutation does have a moderate impact on both folding (ΔΔG 1.0 kcal/mol) and trimer assembly (ΔΔG 1.7 kcal/mol), which together would suggest that this mutation could be damaging, even in the absence of 18-mer formation. However, when considering the mutation within the context of 18-mer formation, S163R is predicted to have an extremely large destabilisation effect (ΔΔG 11.1 kcal/mol). This emphasises the importance of the S163 residue for assembly of the high molecular weight multimer, perhaps explaining the presence of two independent L-ORD mutations affecting this site.

In contrast to the three novel L-ORD mutations, the rare coding changes seen in ExAC are in general predicted to have a milder effect C1QTNF5. Overall, when we consider the total destabilisation, combining monomer, trimer and 18-mer contributions, the pathogenic mutations are significantly more destabilising than the coding variants seen in ExAC (*p* = 0.002; Wilcoxon rank-sum test). However, some ExAC variants, such as V150F, L191P and G229A, are also classed as deleterious or damaging by the online prediction tools and predicted to be destabilising, based on our structural modelling (Fig. 5c; Supplementary Table S1; Supplementary Table S2).

### Mutant C1QTNF5 proteins are unstable *in vitro* and show reduced secretion from cells

**Figure 6:**
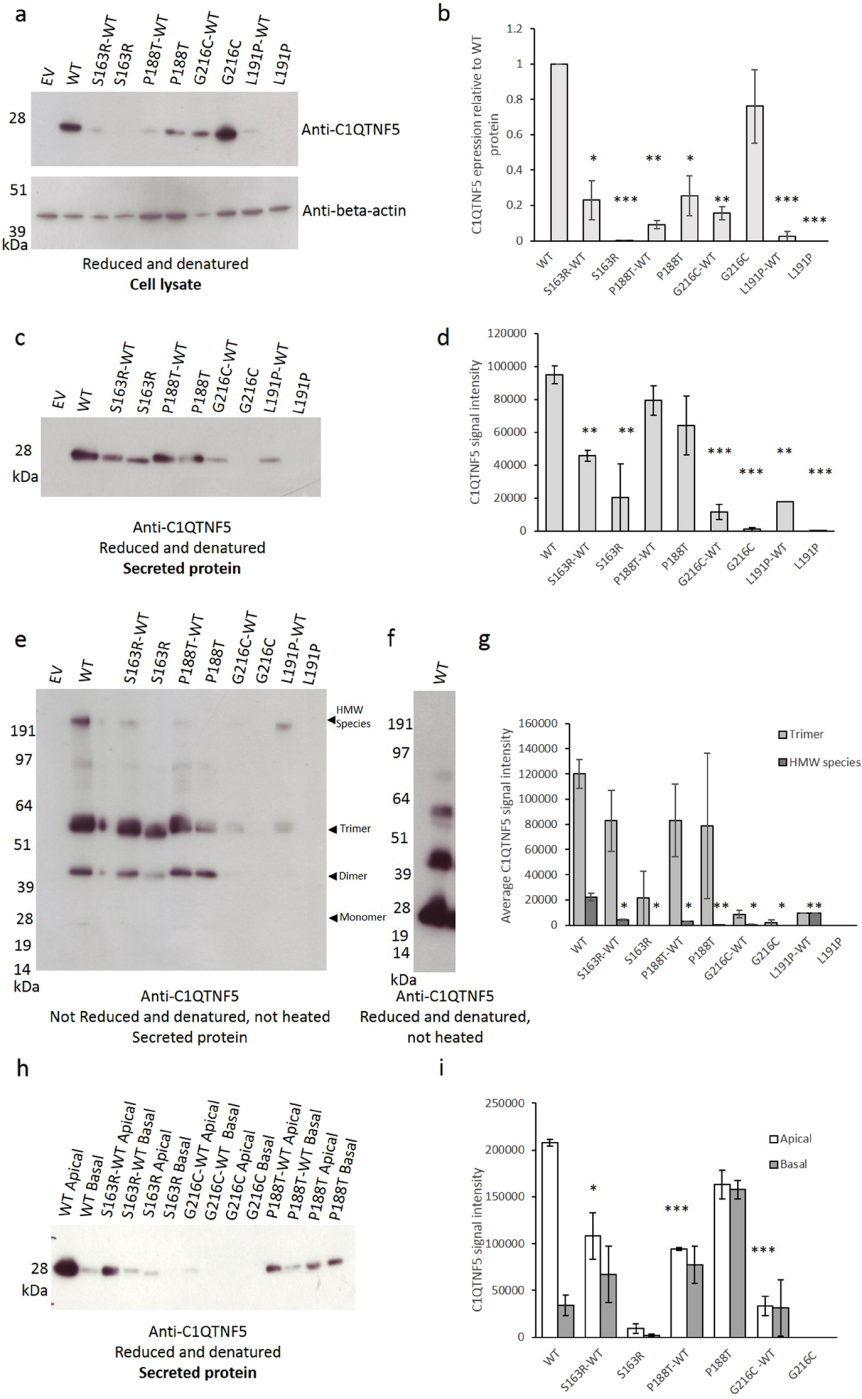
Expression of C1QTNF5 in hTERT-RPE1 cells. (**a**) Recombinant C1QTNF5 is a 28 kDa monomer in reduced and denatured transiently transfected hTERT-RPE1 cell lysates, immunoblotted for C1QTNF5, with beta-actin as a loading control. (**b**) densitometric analysis shows that mutant proteins are expressed significantly less than WT C1QTNF5 intracellularly, after normalisation to beta-actin. *p < 0.05,**p < 0.01, ***p < 0.001 (T-test). (**c**) Secreted C1QTNF5 is a monomer of 28 kDa. Mutant C1QTNF5 shows varying levels of secretion, quantified by densitometry in (**d**). (**e)** Secreted C1QTNF5 forms complexes, including trimers and higher order structures, that are electrophoretically stable under non-reducing conditions (**f**) The high molecular weight complex of C1QTNF5 is held together by disulphide bonds and is not observed under reducing conditions. Complex formation observed in (**e**) is disrupted by the pathogenic mutations, quantified by densiotometry in (**g**). Statistically significant differences in the proportion of total C1QTNF5 that forms stable HMW complexes are indicated. (**h**) Mutation of C1QTNF5 results in decreased apical secretion and a shift towards a greater proportion of C1QTNF5 secreted from the basal surface of transiently transfected cells grown on a transwell membrane, quantified by densitometry in (**i**). Representative blots from a minimum of three separate transfections are shown, error bars show standard error of mean (s.e.m.). Full-length blots are presented in Supplementary Figure 3.

In order to confirm the impact of the new L-ORD mutations on the stability and function of C1QTNF5 *in vitro*, the sequence encoding full-length *C1QTNF5*, including the signal peptide (amino acids 1–15), collagen domain (amino acids 30–98) and gC1q domain (amino acids 99–243; Fig. 4a), was cloned from human retinal cDNA and inserted into a mammalian expression vector. Site-directed mutagenesis was used to engineer the nucleotide changes required to express C1QTNF5 mutants S163R, P188T and G216C, in addition to the ExAC variant L191P, which is predicted to be unstable (Supplementary Table S2). Analysis of hTERT-RPE1 cells transiently expressing mutant or wildtype C1QTNF5 showed striking differences in protein expression, stability and secretion (Fig. 6), without affecting cell viability, cell adhesion or membrane integrity (Supplementary Figure 1). In denaturing polyacrylamide gel electrophoresis (PAGE) analyses of both cell lysates and secreted proteins, wildtype and mutant C1QTNF5 were each detected as monomeric species, with a molecular weight of 28 kDa (Fig. 6a,c). However, consistent with the computational modelling of L-ORD mutations, the mutant proteins behaved differently to wildtype C1QTNF5. Densitometric quantification of the relative expression levels of C1QTNF5 in cell lysates showed significantly reduced expression of the L-ORD mutants S163R and P188T relative to the wildtype protein, after normalisation using beta-actin as a loading control (Fig. 6a,b). G216C appears to be retained intracellularly, and is detected strongly in cell lysate (Fig. 6a) but not in the secreted fraction (Fig. 6c). The results were similar for denaturing PAGE analysis of secreted S163R and G216C proteins, which showed substantially reduced secretion, but the results for the P188T mutant were unexpected. In this mutant there was no significant reduction in P188T protein secreted, relative to the wildtype, despite the significant (p < 0.05) reduction in P188T expression intracellularly. The P188T protein therefore appears to be effectively secreted at levels equivalent to the wildtype (Fig. 6c,d), arguing against a simple haploinsufficiency mechanism in L-ORD.

The assembly of trimers and higher order multimers of C1QTNF5 was analysed for the secreted proteins under non-reducing PAGE conditions in the absence of heating (Fig. 6e), or under reducing PAGE conditions (Fig. 6f). Densitometric quantification confirmed that secretion of the 216C and 163R mutant proteins is greatly reduced, as with the lysates, with highly significant reductions in the amount of C1QTNF5 detected in the conditioned media from transfected cells (Fig. 6e,g). In contrast, the P188T mutant showed similar amounts of monomer, dimer and trimer as the wildtype. However, the amount of high molecular weight multimer, presumed to represent the C1QTNF5 octadecamer^12^, was severely reduced in all three mutants (Fig. 6e,g), and was abolished by preparation of the samples under reducing conditions (Fig. 6f).

### Mutant C1QTNF5 exerts dominant negative effects on wild type protein

hTERT-RPE1 cells were used to transiently co-express both mutant and wildtype C1QTNF5, in order to simulate the situation in L-ORD heterozygotes. In agreement with the computational predictions, G216C, when co-expressed with the wildtype (WT) protein, appeared to destabilise the wildtype, such that less than 20% of the expected signal was detected for G216C-WT protein by western blot (Fig. 6a,b). Similarly, when either S163R and P188T were co-expressed with wildtype protein, the amounts of C1QTNF5 in lysates were greatly reduced (Fig. 6a,b), suggesting a dominant negative disease mechanism. Secreted proteins were also analysed and showed that while S163R or G216C co-expressed with wildtype reduces the amount of secreted C1QTNF5, this is not the case for P188T which does not significantly reduce C1QTNF5 secretion compared with wildtype (Fig. 6c,d).

In non-reducing PAGE analysis of secreted proteins following co-expression of wildtype and mutants in hTERT-RPE1 cells, the monomer, dimer and trimer bands were present and apparently stable and present in the expected amounts but the high molecular weight (HMW) species was less abundant or absent when each L-ORD mutant was co-expressed with wildtype (Fig. 6e,g). The HMW species accounted for only 1.5% of total C1QTNF5-specific signal on the western blot, compared to 15% for the wildtype protein, when wildtype protein was co-expressed with S163R or P188T mutants. A similar reduction of the HMW species was found for co-expressed wildtype and G216C. These results are again consistent with a dominant negative effect and may reflect an inability of heterotrimers containing G216C, S163R or P188T subunits together with wildtype to assemble into higher order structures. The L191P variant identified in the ExAC database was expressed in hTERT-RPE1 cells, and significantly reduced the expression of C1QTNF5 relative to wildtype (p = 0.0018; Fig. 6c,d), in agreement with structural modelling of this variant (Supplementary Table S2, Fig. 5b,c). In contrast to the L-ORD mutants, when L191P was co-expressed with wildtype C1QTNF5, the relative abundance of the secreted HMW species increased to 50% of total protein detected (Fig. 6e,f) despite a significant reduction in both the total amount of C1QTNF5, and trimer formation, detected on the western blot. This suggests that that L191P variant, which has not been associated with L-ORD, does not exert the same dominant negative effect as the L-ORD mutations upon formation of the HMW species in heterozygous individuals.

### Mutant C1QTNF5 proteins show altered polarity of secretion

Following a recent report that the S163R mutation leads to secretion of C1QTNF5 from the baso-lateral rather than apical surface of RPE cells in the mouse^19^, we investigated whether the novel L-ORD mutations alter the route of secretion, in addition to affecting stability and amount of C1QTNF5 secreted. Conditioned medium was therefore collected from the apical and basal surfaces of transiently transfected hTERT-RPE1 cells maintained in transwell plates. This confirmed that wildtype C1QTNF5 is predominantly secreted from the apical rather than the baso-lateral surface (Fig. 6h,i). The strongest redistribution towards the basal surface of the transfected cells was observed following transfection of a single plasmid encoding the P188T mutant (Fig. 6h,i). All three L-ORD mutant proteins, when co-expressed with wildtype to recapitulate the heterozygous genotype in patients, result in a striking redistribution of total C1QTNF5 towards being secreted from the baso-lateral surfaces as well as apical surfaces of hTERT-RPE1 cells (Fig. 6h,i). As shown in Supplementary Figure 2, this is not observed in co-transfections of the WT with GFP or the EV control, but arises as a response to the expression of L-ORD mutant C1QTNF5 proteins in hTERT-RPE1 cells grown on a transwell insert.

## Discussion

A single founder mutation in C1QTNF5 (S163R) is the only mutation reported to cause L-ORD to date^5^. Here, we report the identification of three novel pathogenic variants in *C1QTNF5* in independent L-ORD families, and show firstly that these variants destabilise the protein by disrupting intramolecular and intermolecular interactions that are crucial for maintaining correct folding and assembly. We also show that the polarity of secreted C1QTNF5 is altered, towards a baso-lateral rather than apical distribution, in each of the mutants. Finally, we report the results from *in vitro* expression analyses that suggest a dominant negative disease mechanism, whereby the mutant protein oligomerises with wildtype and in so doing destabilises the protein and reduces the amount of hetero-oligomer both in cell lysates and secreted proteins.

The crystal structure of wild-type C1QTNF5 gC1q domain trimer confirmed that, as for other members of the C1q/TNF family, protomers of C1QTNF5 interact to form a trimer^12^. Size exclusion chromatography and electron microscopy further suggested that it forms a bouquet-like octadecamer, consisting of six trimers^12^. This high molecular weight assembly, similar to the structure of adiponectin and complement component C1q, has been proposed as the functional unit for C1QTNF5, acting in extracellular matrix adhesion and cell-to-cell adhesion^12^. There are varying reports on the requirement for intermolecular disulphide bonds, between cysteine residues in the collagen-like domain, to form the C1QTNF5 multimers, but there are undoubtedly numerous non-covalent intramolecular interactions required to allow correct folding of the C1QTNF5 monomer, as well as intermolecular bonds to maintain the assembly of the trimer and higher order structures^11,12,20^.

The L-ORD and ExAC missense substitutions were mapped onto the monomeric gC1q domain structure of C1QTNF5 (Fig. 5a). The ExAC variants were uniformly distributed across the globular gC1q domain of C1QTNF5, but all three L-ORD mutants are located within this domain, consistent with it having an important role in structure or function. *In silico* structural analysis predicted that the three L-ORD mutations, G216C, P188T and S163R, all result in energetically unfavourable changes to C1QTNF5 (Fig. 5c). Destabilising intramolecular interactions, such as those predicted by the alteration of glycine at position 216 for cysteine, introduce steric clashes with the amino acids adjacent in the tertiary structure (V159, A162, S163 and L164), and affect stability of the monomer. This can be seen at the protein level, when the G216C mutant is expressed in hTERT-RPE1 cells. Mutant G216C appears to accumulate intracellularly (Fig. 6a) but there is minimal secretion of this mutant into the conditioned medium (Fig. 6c). When the mutant protein is co-transfected with wildtype C1QTNF5, only a small amount of C1QTNF5 is secreted, less than 10% of the protein secreted when only wildtype C1QTNF5 is present (Fig. 6c,d). The latter supports a dominant negative mechanism in which the wildtype protein is depleted by its association with the unstable mutant. The S163R and P188T mutants are less destabilising compared to wildtype when co-transfected into cells, consistent with their less energetically unfavourable effects upon protein folding (Fig. 5c). Previous work found that C1QTNF5 S163R was not secreted by transfected cells^21,22^, which was confirmed in this study (although we did not observe an intracellular aggregate as previously reported). By contrast with G216C and S163R, the P188T mutant appears to be secreted at similar amounts to the wildtype, and to form homotrimers and heterotrimers when expressed with the wildtype protein. This appears to preclude haploinsufficency as a mechanism for L-ORD, which was previously suggested^22^. However, despite having apparently equivalent amounts of C1QTNF5 secreted by the transfected cells, P188T alone does not form the high molecular weight complex observed when the wildtype protein is co-expressed (Fig. 6e). There is no significant difference in the proportion of trimers formed when P188T and S163R are co-expressed with the wildtype protein, suggesting that heterotrimers can form, in contrast to the HMW species. Although mutant and wildtype proteins differing by only a single amino acid cannot be electrophoretically resolved, our results are consistent with previous work showing that wildtype and mutant C1QTNF5 can multimerise *in vitro* ^22^.

The significant reduction in formation of the high molecular weight C1QTNF5 species (Fig. 6e), predicted to be the functional unit^12^, when the C1QTNF5 mutants are co-expressed with wildtype, suggests a dominant negative mechanism in L-ORD. This implies a situation where a mutant antagonises the wildtype in a dominant manner. This mechanism is suggested by the observed reductions in wildtype, both for intracellular and secreted proteins, following co-expression (Fig. 6a,e). It suggests that heterotrimers formed between wildtype and mutant monomers may be unable to assemble into higher order structures, and that these residues have a key role in their assembly. Tu and Palczewski^12^ found that an intermolecular network of hydrogen bonds was important for the assembly of the higher order structures, consisting of clustered C1QTNF5 trimers. This intermolecular network of bonds involves the hydroxyl group of S163, and the side chain of K187 from an interacting C1QTNF5 monomer. Our results are consistent with the possibility that mutation of S163, or displacement of the K187 residue by mutation of the adjacent P188, have an equivalent effect on destabilising or preventing the formation of high molecular weight C1QTNF5 assemblies.

Mutation of C1QTNF5 may affect epitope recognition within the oligomeric species of C1QTNF5 by altering the access and affinity of the anti-C1QTNF5 antibody used in this study. To minimise this possibility, a polyclonal antibody that is likely to recognise multiple epitopes was used. If the higher order structures containing mutant protein subunits do form in an equivalent amount to the WT alone but create novel conformational epitopes that are not recognised by the polyclonal antibody, we would expect to see no difference in total C1QTNF5 detected when the complexes are reduced and denatured to expose only linear epitopes. This is not the case, and each L-ORD mutant has a distinct impact on the C1QTNF5 signal detected in cell lysates and secreted protein (Fig. 6).

Taken in isolation, the lack of HMW species of C1QTNF5 in the presence of either S163R or G216C mutants may have suggested that insufficient amounts of protein contributes to the lack of HMW species. However, by studying two additional mutants, it appears that this is not the only reason for the diminished HMW species of C1QTNF5. The P188T mutant (either alone, or in combination with the WT) is secreted in roughly equivalent amounts to the WT protein (Fig. 6c,d), and yet, there is still a significant reduction of HMW species detected (Fig. 6e and g). Conversely, the L191P mutant identified in ExAC significantly decreases total C1QTNF5 expression and secretion when co-expressed with the WT (Fig. 6a–d), to a similar degree as G216C co-expressed with WT C1QTNF5. However, the HMW species is not diminished to the same degree by L191P and G216C (Fig. 6e). Taken together with our computational modelling (Fig. 5), these data suggest that the higher order structures are not less abundant solely as consequence of insufficient amounts of C1QTNF5 protein. Rather, these data suggest that the L-ORD mutants may share a common pathogenic mechanism, whereby the HMW species of C1QTNF5 is destabilised when wildtype and mutant protein are co-expressed (such as would occur in heterozygous individuals).

Directional secretion of proteins by the polarised RPE monolayer contributes to retinal homeostasis and function, and incorrect secretion of structural or regulatory components of the extracellular matrix has previously been associated with the development of age-related macular degeneration. A recent report shows that wildtype C1QTNF5, expressed in mouse RPE from an adeno-associated virus (AAV) vector, is primarily secreted from the apical surface, while the S163R mutant protein is rerouted to the basolateral surface^19^. Consistent with this, we observed a striking alteration in the direction of secretion of mutant C1QTNF5 when co-expressed with wildtype, from predominantly apical expression with wildtype alone, towards greater basolateral expression (Fig. 6h, i). To varying degrees, all three L-ORD mutations result in this shift towards basolateral secretion. A similar re-routing has been described for the Alzheimer’s disease-associated “Swedish” mutation in the amyloid precursor protein (APP), resulting in apical mis-sorting of 20% of APP following beta-secretase cleavage^23^. hTERT-RPE1 cells have been reported to differentiate and polarise when grown on transwell inserts^24^, sharing common features with polarised RPE cells *in vivo*. The mechanism underlying mis-sorting of C1QTNF5 in either hTERT-RPE1 cells or in mouse RPE^19^ is unclear, and it remains to be tested whether this is a consequence of altered RPE cell polarity, either as a cause or consequence of cellular dysfunction. Following transient transfection, the RPE cells used in our study were not maintained in culture for long enough to observe sub-RPE deposit formation, as was seen in mice at 9 months after AAV-C1QTNF5 S163R injection^19^. It is tempting however to speculate that secretion of misfolded and unstable C1QTNF5 to the basolateral surface of the RPE may contribute to the accumulation of drusenoid deposits characteristic of L-ORD^4^.

Following the identification of three novel pathogenic variants in C1QTNF5, we have shown that these mutations appear to act in a dominant negative manner, reducing the amount of available wildtype protein in the heterozygote, particularly the high molecular weight form. The identification of two independent mutations causing the same amino acid substitution (S163R) suggest that this residue is particularly important for protein assembly, as predicted in the modelling of the higher order structure (Fig. 5b,c). This type of codon specificity is found in other dominant negative mutations, as in the *ITPR1* ^25^ and *TP53* genes^26^. A dominant negative mechanism is likely to affect one or more of the following: (1) folding and stability of the C1QTNF5 monomer, (2) assembly and stability of the C1QTNF5 trimer or HMW species, (3) secretion of C1QTNF5 from the cell and resulting mis-localisation of the protein. These factors are likely to result in impaired function of assembly-competent C1QTNF5 trimers, although the physiological role of C1QTNF5 remains to be determined. Similar mechanisms have been described in other autosomal dominant macular dystrophies, including Stargardt-like macular dystrophy, where heterozygous mutations in *ELOVL4* alter the subcellular localisation of the protein^27^. This is also seen in Sorsby’s fundus dystrophy, where heterozygous mutations in *TIMP-3* cause accumulation of degradation-resistant TIMP-3 multimers in the extracellular matrix^28^. In both diseases, which have striking clinical similarities to L-ORD, heterozygous mutations have dominant negative effects on protein function, leading to RPE dysfunction.

Recently it was observed that dominant negative mutations tend to be structurally milder than other recessive or dominant mutations, at least in oligomeric transmembrane channels^25^. This is consistent with the fact that a dominant negative mechanism essentially requires some assembly to occur, in order for the mutated protein to inhibit the activity of the wild type protein. This contrasts with the results here, where all three pathogenic *C1QTNF5* mutations are all highly destabilising. However, it is important to note that, in addition to the trimeric and higher-order contacts facilitating oligomerisation of the globular gC1q domain, there is also oligomerisation via the collagen domain, which may not be affected by these mutations. Thus, the dominant negative mechanism seen here may be reliant on the fact that the C1QTNF5 protein possesses two separate domains involved in oligomerisation.

This description of allelic heterogeneity in *C1QTNF5* suggests that there may be additional pathogenic variants in *C1QTNF5*, as well as in other genes, that lead to L-ORD. No phenotypic information is available for the individuals in ExAC, but rare variants, such as p.V150F (c.448G > T), recorded in a single heterozygous individual of European ancestry (minor allele frequency 0.000008399), may have similar pathological consequences as the L-ORD variants, based on the predicted impact on intramolecular interactions and protein stability shown in Supplementary Table S2 and Fig. 5b,c). Other rare variants in C1QTNF5, such as p.L191P (c.572T > C), recorded in 2 individuals of African descent out of over 5,000 sequenced individuals (minor allele frequency 0.0001938) may act to reduce the amount of C1QTNF5 secreted, but have limited impact upon the formation of HMW species in heterozygous individuals (Fig. 6c,d). This study highlights the importance of incorporating quaternary structural information in order to understand the impact of pathogenic variants on C1QTNF5 structure and function, and in the identification and prioritisation of causal variants amongst other rare, non-synonymous amino acid changes.

Gene augmentation therapy by subretinal injection of adeno-associated viral vectors has resulted in visual improvement in an autosomal recessive retinal dystrophy^29,30^. In L-ORD, the disease mechanism appears to be different however and potentially less amenable to this approach. The provision of additional C1QTNF5 protein to the RPE may only result in a marginal increase in functional wildtype due to the proposed dominant negative effects of the mutants, which appears to tie up wildtype protein in structurally sub-optimal and unstable multimers. Alternative approaches such as RNA interference^31^ or gene editing may therefore have advantages.

## Methods

### Patient ascertainment, diagnoses and DNA samples

Blood or saliva samples were obtained from two families affected by autosomal dominant L-ORD, and 23 unrelated suspected L-ORD cases identified after referral of the probands for ophthalmological examination. DNA was extracted using a standard protocol, and all samples genotyped for the previously described S163R mutation in *C1QTNF5* ^5^. The study was approved by the Lothian Research Ethics Committee (reference 1702/95/4/192) and the institutional review board at the University of Pennsylvania, and was performed in accordance with NHS Lothian and HIPAA guidelines. Informed consent was obtained and all procedures adhered to the tenets of the Declaration of Helsinki.

For family 1, colour fundus photography was performed using the TRC-501X (Topcon medical systems, New Jersey, U.S.A). Images were captured on a Nikon D90 (Nikon, Japan). Confocal scanning laser ophthalmoscope (SLO) images were obtained for fundus autofluorescence (AF) (wavelength = 480 nm) using the Spectralis (Heidelberg Engineering, Heidelberg, Germany). Spectral domain OCT (SD-OCT) imaging was carried out using a Heidelberg Spectralis SD-OCT (Heidelberg Engineering, Heidelberg, Germany). Dark adaptometry (DA) was performed either using a portable two colour dark adaptometer using a previously published protocol^32^ or using a visual stimulus generator (VSG 2/5, Cambridge Research Systems, Rochester, UK) to generate stimuli and using Visual Psychophysics Engine software (Cambridge Research Systems, customized by NRAP) and presented on a calibrated and gamma-corrected high-resolution CRT monitor (Sony GDM-F500R, Tokyo, Japan) using a previously published protocol^33^. For microperimetry, the MP1 was used (Nidek, Gamagori, Japan). A 4-2 double staircase threshold strategy was used using a Goldman size III target, which was presented for 200 ms. A monochromatic background was set at 2.27 cd/m^2^ (=4 asb).

For family 2, *en face* imaging was performed with a confocal SLO (HRA2, Heidelberg Engineering, Heidelberg, Germany) using near infrared reflectance mode. Horizontal cross-sectional images were obtained with SD-OCT (RTVue-100; Optovue, Inc., Fremont, CA). Chromatic dark- and light-adapted static perimetry was used to quantify rod- and cone-mediated visual function across the visual field^4^.

Unaffected individuals aged over 50 years of age were clinically examined and also showed normal two-colour dark adaptometry results. Individuals aged under 50 years were assumed to be of status unknown.

An additional 23 L-ORD patients were clinically characterised as described for family 1, and used for additional *C1QTNF5* gene mutation screening.

### Screening *C1QTNF5* for mutations

The coding and regulatory regions of *C1QTNF5* were analysed by direct sequencing, using primers as previously described (Hayward *et al*.^5^). In family 1, the G216C mutation was confirmed by restriction digestion using HpyCh4V (NEB) following PCR using the primers CTRP5x3AF (5′GAGGGGTACGGTGACCTTAGA3′) and CTRP53′UTRBR (5′ACCATGATCCCAGAAACAGG3′), to generate fragments of 439, 363, 129 and 120 bp in homozygous G (p.G216) samples, and 439, 363, 129, 94 and 26 bp for the mutant G > T (p.C216) allele. Restriction digest screening using HpyCh4V was also performed 351 unrelated control samples from the Scottish population and 244 Scottish age-related macular degeneration (AMD) cases, to validate the mutation. No digests were available for genotyping p.P188T or the novel p.S163R change.

### Bioinformatics

The Exome Aggregation Consortium (ExAC) (http://exac.broadinstitute.org) database was interrogated to validate the likelihood of these non-synonomous coding changes in *C1QTNF5* being pathogenic variants^13^. Rare variants in *C1QTNF5* that were in the ExAC database were subsequently included in *in silico* prediction of the consequences of non-synonymous amino acid changes upon the protein. Orthologous gene sequences were downloaded from ensembl (www.ensembl.org/), and amino acid sequences aligned using ClustalOmega (www.ebi.ac.uk/Tools/msa/clustalo/) in order to identify conserved residues. Conservation scores for each residue were calculated using PhyloP and PhastCons.

### Prediction of effect of mutation and structural analysis

Online prediction tools Mutationtaster (http://www.mutationtaster.org/), PROVEAN and SIFT (http://provean.jcvi.org/genome_submit_2.php) and PolyPhen-2 (http://genetics.bwh.harvard.edu/pph2/) were used to predict the impact of non-synonomous amino acid substitutions on C1QTNF5.

The effects of each amino acid substitution on protein folding and assembly were predicted using FoldX^34^. The locations of the three pathogenic mutations, as well as 32 missense variants present in ExAC^13^, were located in the homotrimeric crystal structure of the human C1QTNF5 gC1q domain (PDB ID: 4NN0)^11,12^. The 18-mer assembly was constructed using contacts present in the asymmetric unit of this structure, essentially as was previously described^12^. The predicted effect on protein folding is the FoldX-calculated change in stability induced by an amino acid substitution for the monomeric subunit in isolation from the rest of the complex. The predicted effect on trimer assembly is the difference between the FoldX-calculated change in stability for the trimer and the monomeric subunit in isolation. Finally, the predicted effect on higher-order assembly is the difference between the change in stability for the 18-mer and the trimer. When considering the full complex, only a single subunit from the trimer or 18-mer was mutated, reflecting the heterozygous nature of the mutations. The FoldX “RepairPDB” function was run prior to mutations. Ten FoldX replicates were performed for each mutation, and the average was used.

### DNA constructs

Full-length wildtype *C1QTNF5* was amplified by PCR from human retinal Marathon-Ready cDNA (BD Biosciences) using gene-specific *att*B-flanked PCR primers (Forward 5′-GGGG ACAAGTTTGTACAAAAAAGCAGGCTTCACCATGAGGCCACTCCTCGT-3′, Reverse 5′-GGGGACCACTTTGTACAAGAAAGCTGGGTCagcaaagactggggagct-3′). The *C1QTNF5 att*B-flanked PCR product was gel purified (Qiagen) and used in a Gateway BP recombination reaction with the donor vector pDONR221, and then shuttled into pDEST/C-SF TAP^35^ via Gateway LR gateway recombination as described by the manufacturer (ThermoFisher Scientific). Recombination reactions were used to transform TOP10 *E*. *coli* (Invitrogen), and selection performed using kanamycin (50 μg/mL) and ampicillin (100 μg/mL) for the entry vector and the destination vector respectively. Plasmids were isolated using a plasmid miniprep kit (Qiagen), and the cDNA insert was verified by sequencing. Mutations were made in the destination vector using the QuikChange Site Directed mutagenesis II kit (Stratagene), according to the manufacturer’s protocol and using the following primers for mutagenesis (p.S163R (c.489C > G) anti-sense 5′-cagatcaaactgcagcctggcccggtagac-3′, sense 5′-gtctaccgggccaggctgcagtttgatctg-3′, p.P188T (c.562C > A), anti-sense 5′-agagcgaggctgtcttgggccaccc-3′, sense 5′-gggtggcccaagacagcctcgctct-3′, p.G216C (c.646 G > T) anti-sense 5′-gctggcatagatgcaaatgtagtcacccacaccc-3′, sense 5′-gggtgtgggtgactacatttgcatctatgccagc-3′, and p.L191P (c.572T > C), anti-sense 5′-ccccccccgagggcgaggctggc-3′, sense 5′-gccagcctcgccctcgggggggg-3′. The mutations were confirmed by sequencing.

### Cell culture and transfection

hTERT-RPE1 cells were grown in Dulbecco’s Modified Eagle Medium: Nutrient Mixture F-12 (DMEM/F-12; Gibco) containing 15mM HEPES, 2mM L-Glutamine and 0.348% sodium bicarbonate, supplemented with 10% fetal calf serum (FCS) and penicillin/streptomycin. Cells were incubated at 37 °C in a humidified CO_2_ incubator. For each transfection reaction, 5 × 10^6^ cells were mixed with 5 µg of plasmid DNA. For co-transfections of wildtype (WT) and mutant plasmids, cells were mixed with 2.5 µg of each plasmid in the following combinations: pDEST C1QTNF5-CTAP (WT) plus pDEST C1QTNF5 S163R-CTAP, pDEST C1QTNF5 P188T-CTAP, pDEST C1QTNF5 L191P-CTAP or pDEST C1QTNF5 G216C-CTAP. Co-transfections of 2.5 µg wildtype (WT) with 2.5 µg pDEST CTAP empty vector (EV) or with 2.5 µg pmaxGFP were also performed. Microporation was performed using the Neon transfection system (ThermoFisher Scientific) as described by the manufacturer. Microporation parameters were 1500 V/20 ms/1 pulse. Following microporation, each reaction was seeded into a 10cm plate containing antibiotic-free DMEM/F-12 supplemented with 15mM HEPES, 2mM L-Glutamine, 0.348% sodium bicarbonate and 10% FCS. In some experiments, transfected cells were seeded on Transwell filters (Corning) and allowed to form a confluent monolayer. Cells were incubated at 37 °C in a humidified CO_2_ incubator for 24 hours, then washed in Phosphate-buffered Saline (PBS), and media was changed to serum-free for a further 48 hours. Conditioned media was collected, and centrifuged at 1200 rpm for 10 minutes to remove cell debris, then concentrated approximately 10-fold using a Centriprep centrifugal filter device (Amicon; 3kDa MWCO) at 3000 × *g* at 4 °C. Cells were washed with PBS, and cell lysates were prepared in RIPA buffer (150 mM NaCl, 50 mM Tris pH 8, 1% NP-40, 0.5% sodium deoxycholate, 0.1% SDS). Transfections were performed a minimum of three times for each plasmid combination.

### Immunoblotting

For reducing gels, samples containing equal amounts of total protein were mixed with 1 × Reducing Agent (Invitrogen) and 4 × NuPAGE LDS (lithium dodecyl sulfate) Sample Buffer (Invitrogen). Where stated, samples were also denatured by heating to 80 °C for 15 minutes. Samples run under non-reducing conditions were mixed with 4 × NuPAGE LDS Sample Buffer and loaded directly on the gel, without heating. Gel electrophoresis was performed using 4–12% Bis-Tris NuPAGE gels (Invitrogen) run in 1 × MOPS (3-(N-morpholino)propanesulfonic acid; Invitrogen). Proteins were transferred to Hybond-P PVDF membranes (GE Healthcare) in 1 × NuPAGE Transfer Buffer (Invitrogen) containing 20% methanol (Fisher). Membranes were blocked in 5% non-fat milk in PBST (Phosphate-buffered Saline-Tween 20; 3.2 mM Na_2_HPO_4_, 0.5 mM KH_2_PO_4_, 1.3 mM KCl, 135 mM NaCl, 0.05% Tween 20, pH 7.4) before being incubated with rabbit anti-C1QTNF5 IgG (Strategic Diagnostics, Inc) or mouse anti-β actin IgG (T5441, Sigma), followed by anti-rabbit or anti-mouse IgG horseradish peroxidase (HRP)-conjugated secondary antibodies. Protein bands were visualised by addition of Enhanced Chemiluminescence (ECL) Western blotting detection reagents (GE Healthcare) and exposure of the membrane to Kodak BioMax XAR film (Sigma). Films were developed using a phosphorimager X-ray machine (Konica Minolta). Densitometry analysis of scanned films was performed using ImageStudioLite software (Li-Cor). Statistically significant differences between a minimum of three separate experiments were assessed by t-test (GraphPad QuickCalcs).

### Data Availability Statement

The datasets supporting the conclusions of this article are included within the article and supplementary files.

## Electronic supplementary material

Supplementary Information
