# Supplementary material for: Novel pathogenic mutations in C1QTNF5 support a dominant negative disease mechanism in late-onset retinal degeneration
Source: Sci Rep. 2017 Sep 22;7:12147. doi: 10.1038/s41598-017-11898-3 (PMC5610255; doi:10.1038/s41598-017-11898-3)
Supplement: Supplementary file 1 — Supplementary Information [file 41598_2017_11898_MOESM1_ESM.pdf]

Supplementary Information for:

Novel pathogenic mutations in *C1QTNF5* support a dominant negative disease mechanism in late-onset retinal degeneration.

Chloe M. Stanton[1*], Shyamanga Borooah[2,3], Camilla Drake[1], Joseph A. Marsh[1], Susan Campbell[1], Alan Lennon[1], Dinesh C. Soares[1], Neeru A. Vallabh[4,5], Jayashree Sahni[5,6], Artur V. Cideciyan[7], Baljean Dhillon[3,8], Veronique Vitart[1], Samuel G. Jacobson[7], Alan F. Wright[1], Caroline Hayward[1]

Author Affiliations

[1]Medical Research Council Human Genetics Unit, Medical Research Council Institute of Genetics and Molecular Medicine, University of Edinburgh, Edinburgh, United Kingdom

[2]Medical Research Council Centre for Regenerative Medicine, University of Edinburgh, Edinburgh, United Kingdom

[3]Princess Alexandra Eye Pavilion, Edinburgh, United Kingdom

[4]St.Paul's Eye Unit, Royal Liverpool Hospital, Liverpool, United Kingdom

[5]Department of Eye and Vision Sciences, University of Liverpool, Liverpool, United Kingdom

[6]Roche Pharma Research and Early Development, Roche Innovation Center Basel, F. Hoffmann-La Roche Ltd, Basel, Switzerland

[7]Scheie Eye Institute, University of Pennsylvania, Philadelphia, Pennsylvania, USA

[8]Centre for Clinical Brain Sciences, School of Clinical Sciences, University of Edinburgh, Edinburgh, United Kingdom

*Corresponding author (email: chloe.stanton@igmm.ed.ac.uk).

Supplementary Methods

MTT Assay

Cell viability and proliferation was assessed using the 3-(4,5-dimethylthiazol-2-yl)-2,5-diphenyltetrazolium bromide (MTT) assay. After transfection, 0.1x10^6 hTERT-RPE1 cells were seeded per well of 24-well plates. After 24 hours, 0.5 mg/ml MTT (Sigma Aldrich) was added directly to the medium, or cells were washed in PBS and media changed to serum-free for an additional 48 hours before the addition of 0.5 mg/ml MTT for 3 hours at 37°C. MTT crystals were dissolved in MTT Solvent (4 mM HCl, 0.1% Nondet P-40 (NP40) in isopropanol), and absorbance read at 590 nm using a Multiskan Spectrum microplate spectrophotometer (Thermo Scientific). Assays were performed in triplicate, from three separate transfections.

Collagen adhesion assay

24 hours after transfection, hTERT-RPE1 cells were trypsinised, counted and 0.25x10^5 cells/ well used to seed 4 wells of Collagen I-coated 96-well plates (GIbco Life Technologies) that had been blocked with heat-inactivated 1% BSA in PBS for 1 hour at room temperature then washed twice with PBS. Cells were allowed to adhere for 1 hour at 37 °C. For each transfection, additional wells were seeded with 0.5x10^5, 0.25x10^5 and 0.1x10^5 cells for fixing with 5% glutaraldehyde for 20 minutes at room temperature after the 1 hour incubation. Wells were washed three times with PBS to remove non-adherent cells, and adherent cells were stained with 0.1% crystal violet for 1 hour. Wells were washed 5 times in PBS, and then crystal violet was solubilised in 10% acetic acid. Cell adhesion was quantified by measuring absorbance at 570 nm using a Multiskan Spectrum microplate spectrophotometer (Thermo Scientific). Assays were performed in triplicate, from three separate transfections.

GFP co-transfections and Fluorometry

For each transfection reaction, 5 x 10^6 hTERT-RPE1 cells were mixed with 5 µg of plasmid DNA, including 1 µg of pmaxGFP (Amaxa). For transfection of wildtype or mutant plasmids, 4 µg of plasmid was used along with 1 µg of pmaxGFP. For co-transfections of wildtype (WT) and mutant plasmids with GFP, cells were mixed with 2 µg of each plasmid in the following combinations: pDEST C1QTNF5-CTAP (WT) plus pDEST C1QTNF5 S163R-CTAP, pDEST C1QTNF5 P188T-CTAP, pDEST C1QTNF5 L191P-CTAP or pDEST C1QTNF5 G216C-CTAP. Microporation was performed using the Neon transfection system (ThermoFisher Scientific) as described by the manufacturer. Microporation parameters were 1500 V/ 20 ms/ 1 pulse. Following microporation, each reaction was seeded into cell culture plates containing antibiotic-free DMEM/F-12 supplemented with 15mM HEPES, 2mM L-Glutamine, 0.348% sodium bicarbonate and 10% FCS. In some experiments, transfected cells were seeded on Transwell filters (Corning) and allowed to form a monolayer. Cells were incubated at 37°C in a humidified CO_2 incubator for 24 hours, then washed in PBS, and media was changed to serum-free for a further 48 hours. Conditioned media was collected at 24 hours and 72 hours, and GFP in the cells, in conditioned media, and from the apical and basal compartments was measured by fluorometry (excitation 485 nm, emission 535 nm) using a Victor3 plate reader (Perkin Elmer). Transfections were performed a minimum of twice for each plasmid combination.

Immunostaining with ZO-1 and DAPI

24 hours after transfection, 0.25x10^6 cells were seeded on glass coverslips in 6-well plates to form a confluent monolayer for ZO-1 staining. 72 hours after transfection, the cells were washed twice with PBS, and fixed for 15 minutes in 4% paraformaldehyde (PFA). Cells were washed twice with PBS, and then permeabilised with 0.1% Triton X-100 in PBS for 10 minutes, before an additional wash in PBS. Next, the coverslips were incubated in blocking buffer (10% goat serum in PBS) for 1 hour at room temperature. Cells were stained with a primary antibody against zonula occludens (ZO)-1 (1:100, Life Technologies) for 1 hour, washed in PBS, and then incubated with Alexa Fluor 594-conjugated goat anti-mouse secondary antibody (1 :1000) in blocking buffer for 1 hour at room temperature. Cells were counterstained with DAPI. Coverslips were washed 4 times in PBS, and then mounted with Prolong Gold Antifade reagent and epifluorescence imaging was performed at 20X magnification (Zeiss).

Supplementary Table S1: Comparison of the predicted impact of amino acid changes on C1QTNF5 function using online tools.

	PolyPhen2		Provean		SIFT		Mutation Taster	
Mutation	prediction	pph2_prob	SCORE	PREDICTION (cutoff=-2.5)	SCORE	PREDICTION (cutoff=0.05)	Prediction	Probability
S163R	possibly damaging	0.96	-1.57	Neutral	0.01	Damaging	disease_causing	0.94
S163R	possibly damaging	0.96	-1.57	Neutral	0.01	Damaging	disease_causing	0.94
G216C	probably damaging	1.00	-8.62	Deleterious	0.00	Damaging	disease causing	1.00
L191P	probably damaging	1.00	-1.73	Neutral	0.11	Tolerated	disease_causing	1.00
V150F	possibly damaging	0.82	-2.79	Deleterious	0.01	Damaging	disease_causing	0.92
P188T	probably damaging	0.99	-0.56	Neutral	0.15	Tolerated	disease causing	0.99
G229A	probably damaging	1.00	-5.64	Deleterious	0.00	Damaging	disease_causing	1.00
P201S	possibly damaging	0.51	-0.32	Neutral	0.42	Tolerated	disease_causing	1.00
I215T	benign	0.02	2.13	Neutral	0.43	Tolerated	disease_causing	0.52
P148A	probably damaging	0.96	-6.24	Deleterious	0.19	Tolerated	disease_causing	1.00
Q165H	benign	0.00	-1.45	Neutral	0.06	Tolerated	disease_causing	0.98
A162T	benign	0.03	-0.63	Neutral	0.14	Tolerated	polymorphism	0.13
A189V	benign	0.09	-1.46	Neutral	0.66	Tolerated	disease_causing	0.98
I175V	benign	0.00	0.23	Neutral	0.82	Tolerated	polymorphism	0.00
E132K	benign	0.09	-2.51	Deleterious	0.01	Damaging	disease_causing	1.00
E132Q	benign	0.32	-1.71	Neutral	0.38	Tolerated	disease_causing	1.00
A195T	benign	0.28	0.76	Neutral	0.22	Tolerated	polymorphism	0.50
A195G	probably damaging	0.96	-2.78	Deleterious	0.00	Damaging	disease_causing	0.98
S113G	benign	0.02	-1.86	Neutral	0.37	Tolerated	polymorphism	0.03
D224E	benign	0.19	-3.88	Deleterious	0.00	Damaging	disease_causing	0.98

N131K	probably damaging	1.00	-5.99	Deleterious	0.00	Damaging	disease_causing	0.94
V209M	benign	0.09	-1.75	Neutral	0.15	Tolerated	disease_causing	0.75
V139A	benign	0.00	-0.63	Neutral	0.15	Tolerated	polymorphism	0.00
K142R	benign	0.01	-2.11	Neutral	0.23	Tolerated	polymorphism	0.21
R114Q	benign	0.32	-0.54	Neutral	0.08	Tolerated	disease_causing	1.00
G212S	benign	0.03	-0.75	Neutral	0.06	Tolerated	disease_causing	0.98
G183A	benign	0.09	-0.62	Neutral	0.14	Tolerated	disease_causing	1.00
R114W	probably damaging	1.00	-1.82	Neutral	0.01	Damaging	disease_causing	1.00
H135Y	probably damaging	1.00	-5.09	Deleterious	0.00	Damaging	disease_causing	0.91
V139I	benign	0.03	0.3	Neutral	0.52	Tolerated	polymorphism	0.00
S234F	probably damaging	1.00	-2.76	Deleterious	0.04	Damaging	disease_causing	1.00
Y218F	benign	0.29	-1.72	Neutral	0.19	Tolerated	disease_causing	0.98
V128L	possibly damaging	0.48	-1.76	Neutral	0.01	Damaging	disease_causing	0.99
E200Q	benign	0.09	-0.64	Neutral	0.15	Tolerated	polymorphism	0.28
V115L	possibly damaging	0.49	-0.43	Neutral	0.19	Tolerated	polymorphism	0.01
R110L	benign	0.01	3.22	Neutral	1.00	Tolerated	disease_causing	0.65

Supplementary Table S2: Conservation and predicted consequences of pathogenic and ExAC variants upon folding and assembly of the gC1Q domain of C1QTNF5.

Mutation	DNA change	Allele Frequency in ExAC	PhyloP	PhastCons	Total ΔΔG (18-mer)	Standard Deviation	ΔΔG Folding (monomer)	ΔΔG Assembly (trimer)	ΔΔG Assembly (higherorder)
S163R	c.489C>G	0	1.556	1	13.80	2.86	0.97	1.75	11.07
S163R	c.489C>A	0	1.556	1	13.80	2.86	0.97	1.75	11.07
G216C	c.646G>T	0	5.584	1	12.37	0.41	12.01	0.12	0.24
L191P	c.572T>C	0.00001657	3.136	1	8.36	0.03	8.09	0.24	0.02
V150F	c.448G>T	0.000008399	1.142	0.97	7.16	3.90	-0.02	7.18	0.00
P188T	c.562C>A	0	3.797	1	4.14	0.02	2.66	1.47	0.01
G229A	c.686G>C	0.00003354	5.633	1	3.79	0.03	3.76	0.03	0.00
P201S	c.601C>T	0.00001655	5.584	1	3.32	0.01	3.30	0.01	0.00
I215T	c.644T>C	0.00001659	0.496	0.953	2.83	0.01	2.11	0.00	0.73
P148A	c.442C>G	0.000008411	5.445	1	2.71	0.01	2.65	0.06	0.00
Q165H	c.495G>C	0.00004162	2.05	1	2.45	0.07	0.65	2.05	-0.24
A162T	c.484G>A	0.00000834	0.869	0.989	2.15	0.34	0.74	0.03	1.38
A189V	c.566C>T	0.00001658	2.775	1	1.76	0.76	1.69	0.06	0.01
I175V	c.523A>G	0.00001662	0.686	0.221	1.74	0.02	1.89	-0.14	0.00
E132K	c.394G>A	0.000008518	3.632	1	1.59	0.36	0.14	1.50	-0.05
E132Q	c.394G>C	0.00001704	3.632	1	1.29	0.34	0.22	1.12	-0.06
A195T	c.583G>A	0.00000834	0.684	0.976	1.20	1.31	2.20	-1.00	0.00
A195G	c.584C>G	0.000008317	5.584	1	0.79	0.00	0.38	0.41	0.00
S113G	c.337A>G	0.000009376	0.779	1	0.77	0.00	0.74	0.00	0.03
D224E	c.672C>G	0.000008353	2.997	1	0.57	0.37	-0.40	0.84	0.13
N131K	c.393C>A	0.0001959	1.002	1	0.46	0.13	0.57	-0.01	-0.10
V209M	c.625G>A	0.000008279	2.438	1	0.39	0.62	0.47	-0.28	0.20
V139A	c.415G>A	0.000008464	0.289	0.042	0.35	0.03	-0.28	0.00	0.63
K142R	c.425A>G	0.00002531	3.028	1	0.32	0.38	0.50	0.01	-0.19

R114Q	c.341G>A	0.000682	2.728	1	0.25	0.01	0.12	0.00	0.13
G212S	c.634G>A	0.000008287	3.299	1	0.19	0.05	-0.23	-0.01	0.43
G183A	c.548G>C	0.00005809	5.149	1	0.06	0.12	0.08	-0.14	0.12
R114W	c.340C>T	0.000009281	5.409	1	-0.03	0.01	-0.20	0.00	0.16
H135Y	c.403C>T	0.000008492	3.364	0.996	-0.12	0.46	-0.16	0.00	0.04
V139I	c.416T>C	0.00002539	0.07	0.067	-0.14	0.03	-0.22	0.00	0.08
S234F	c.701C>T	0.000008404	5.633	1	-0.33	0.03	-0.39	0.07	0.00
Y218F	c.653A>T	0.00002495	2.981	1	-0.70	0.02	-0.67	0.01	-0.03
V128L	c.382G>C	0.00002571	3.794	1	-0.82	0.03	-0.92	0.11	0.00
E200Q	c.598G>C	0.00002483	2.943	1	-0.83	0.02	-0.53	-0.26	-0.04
V115L	c.343G>C	0.000009138	0.267	0.986	-0.93	0.04	-0.74	0.00	-0.19
R110L	c.329G>T	0.0000491	1.988	1	-2.04	0.31	-2.18	0.03	0.11

Supplementary Figure 1: Expression of C1QTNF5 in hTERT-RPE1 cells does not affect cell viability, cellular adhesion to collagen-coated plates, membrane integrity or expression of the tight junction protein ZO-1. a) Cell viability of hTERT-RPE1 cells was assessed by MTT assay 24 hours and 72 hours (after 48 hours in serum-free media) after transfection. b) Adhesion of transfected cells to Collagen I-coated plates 24 hours after transfection. c) GFP fluorometry of conditioned media collected from the apical and basal chamber of hTERT-RPE1 cells that were co-transfected with pmaxGFP in combination with C1QTNF5 or an empty (EV) expression vectors. Statistically significant differences in GFP detected in the medium compared to EV-transfected cells were assessed by T-test, *p<0.05. d) Immunofluorescent staining of ZO-1 (red) in hTERT-RPE1 cells expressing wildtype, S163R-WT or S163R C1QTNF5 (72 hours after transfection). Nuclei are DAPI-stained (blue). Magnification, 20X.

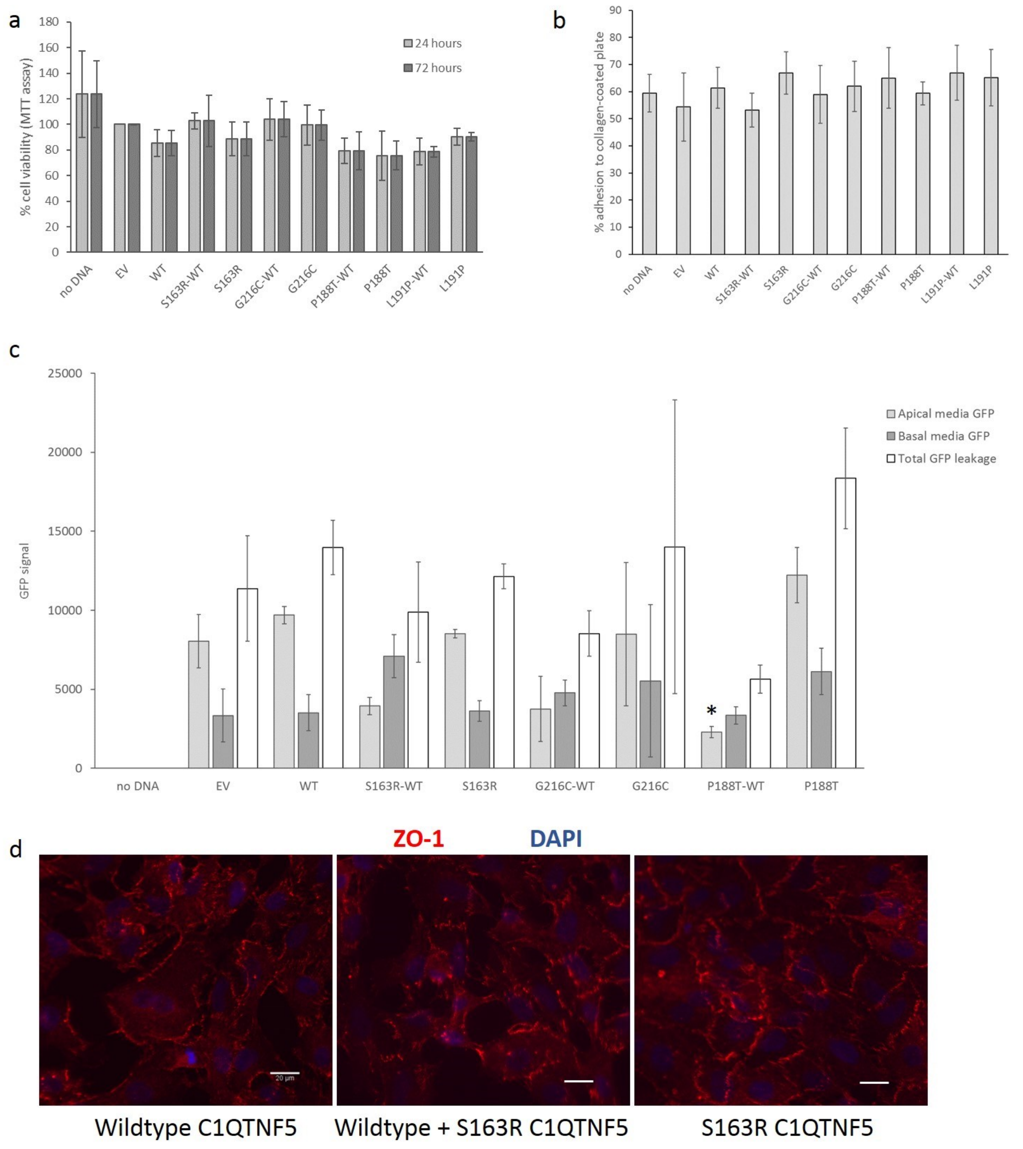

Supplementary Figure 2: Basal redistribution of C1QTNF5 in hTERT-RPE1 cells grown as a confluent monolayer on transwell inserts occurs as a result of mutations in C1QTNF5, rather than as a consequence of co-transfection with two plasmids.

a) Mutation of C1QTNF5 results in a shift towards a greater proportion of C1QTNF5 secreted from the basal surface of transiently transfected cells grown on a transwell membrane. Redistribution of C1QTNF5 is not observed when WT C1QTNF5 is co-transfected with an empty vector (EV) control plasmid, or with a GFP plasmid. b) C1QTNF5 signal was quantified by densitometry and expressed as % of total C1QTNF5 signal detected on the blot. A representative full-length blot from three co-transfections is shown, and error bars represent standard deviation. A significant difference in signal detected at the basal surface is indicated by an asterisk (t-test, p= 0.019).

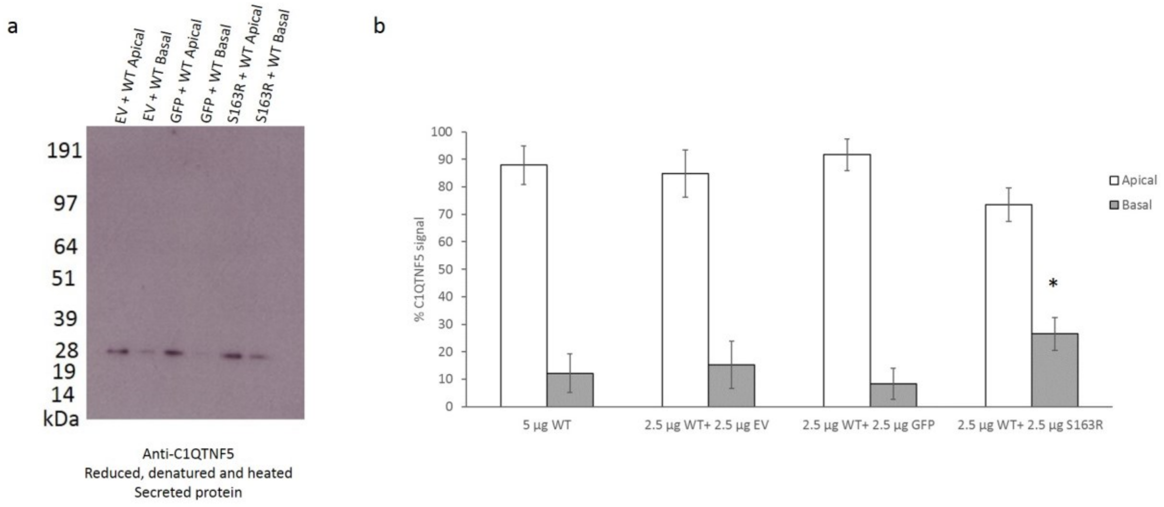

Supplementary Figure 3: Uncropped western blots used in Figure 6

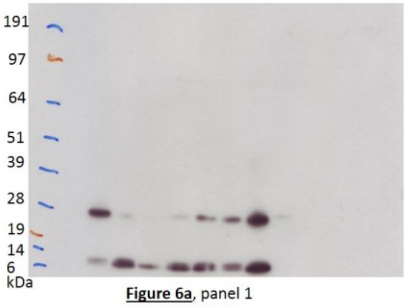

Figure 6a, panel 1
Anti-C1QTNF5
Reduced and denatured
Cell lysate

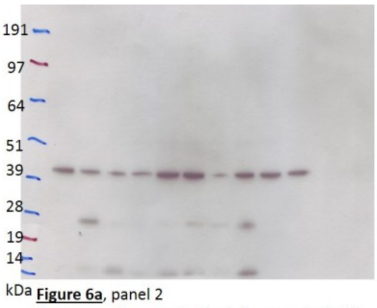

Figure 6a, panel 2
Same membrane as to the left, re-probed with Anti-beta-actin (additional bands are remnants of anti-C1QTNF5 shown to the left)
Reduced and denatured
Cell lysate

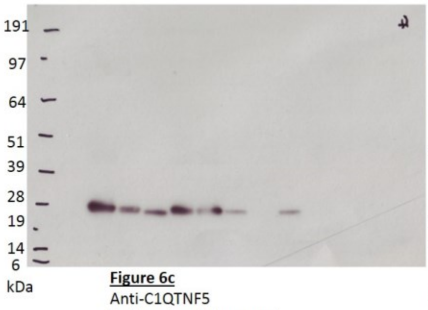

Figure 6c
Anti-C1QTNF5
Reduced and denatured
Secreted protein

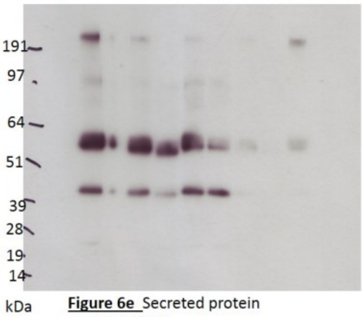

Figure 6e Secreted protein
Anti-C1QTNF5
Not Reduced and denatured, not heated

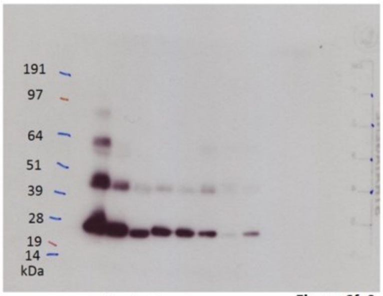

3 minute exposure

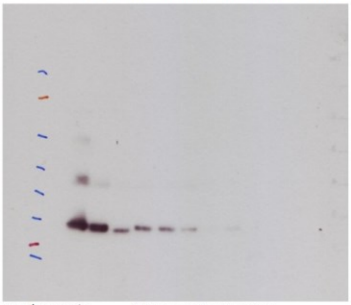

30 second exposure

Figure 6f Secreted protein
Anti-C1QTNF5
Reduced and denatured, not heated

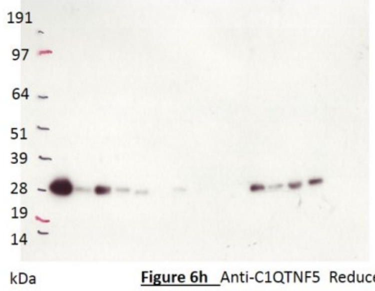

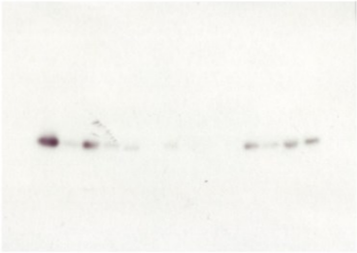

Figure 6h Anti-C1QTNF5 Reduced and denatured **Secreted protein**
3 minute exposure
1 minute exposure
